# Polar Electrophoresis: Shape of Two-Dimensional Maps Is as Important as Size

**DOI:** 10.1371/journal.pone.0030911

**Published:** 2012-01-23

**Authors:** Renato Millioni, Rita Polati, Michele Menini, Lucia Puricelli, Manuela Miuzzo, Paolo Tessari, Enrico Novelli, Pier Giorgio Righetti, Daniela Cecconi

**Affiliations:** 1 Department of Medicine, University of Padova, Padova, Italy; 2 Proteomics and Mass Spectrometry Laboratory, Department of Biotechnology, University of Verona, Verona, Italy; 3 Institute of Neuroscience, National Research Council, Padova, Italy; 4 Department of Public Health, Comparative Pathology and Veterinary Hygiene, University of Padova, Legnaro, Italy; 5 Department of Chemistry, Materials and Chemical Engineering “Giulio Natta”, Politecnico di Milano, Milano, Italy; University of Tor Vergata, Italy

## Abstract

The performance of two-dimensional electrophoresis in conventional gels in Cartesian coordinates (2-DE) vs. polar coordinates (2-PE) is here evaluated. Although 2-DE is performed in much longer Immobiline gels in the first dimension (17 cm) vs. barely 7-cm in 2-PE, an equivalent resolving power is found. Moreover, due to the possibility of running up to seven Immobiline strips in the radial gel format, the reproducibility of spot position is seen to be higher, this resulting in a 20% higher matching efficiency. As an extra bonus, strings of “isobaric” spots (i.e. polypeptides of identical mass with different pI values) are more resolved in the radial gel format, especially in the 10 to 30 kDa region, where the gel area fans out leaving extra space for spot resolution. In conclusion, this novel gel format in the second dimension of 2D gels is seen as an important improvement of this technique, still one of the most popular in proteome analysis.

## Introduction

Two-dimensional electrophoresis (2-DE) has proven to be a key technology in proteomics since the two sequential orthogonal separations are able to deliver maps of several proteins showing changes in the expression level, isoforms and post-translational modifications (PTM). The greatest strength of 2-DE is that protein species differing in PTMs are isolated and can be excised from the gel for further analysis.

2-DE is a technique that has always been subjected to continuous improvements for increasing resolution and experimental reproducibility. In this regards, the introduction of immobilized pH gradients to perform the IEF (instead of the use of carrier ampholytes) [1], the development of soft strips to improve the transfer of proteins from the first to the second dimension [2], [3], the optimization of processing stages, such as the implementation of reduction and alkylation prior to any electrophoretic fractionation [4], the ability to perform multiplexed analyses of different CyDye DIGE labelled samples on the same gel [5] are only some examples taken from a much longer list of 2-DE improvements. More recently, we proposed a new possible upgrade of 2-DE, by changing the shape of the second dimension gel [6]. In this technique, called P-Dimensional electrophoresis (2-PE), the second dimension is performed in a circular crown gel, where the electric field that transports proteins from the first to the second dimension has radial, instead of parallel, lines of force. It has already been shown that this strategy can also improve the separation of spots with similar pI and Mr, compared to classical Cartesian maps obtained using IPG strips of equal length [6] but this comparison was limited to the analysis of a small pool of known proteins since the primary purpose was to demonstrate that the transfer of proteins from first to second dimension was complete, regardless of the gels and electric fields shapes.

To further investigate the comparison of 2-PE *vs.* 2-DE, we report a qualitative evaluation of results obtained with the different approaches, but using Cartesian gels with an area about twice that of radial gels.

## Results and Discussion

**Figure 1:**
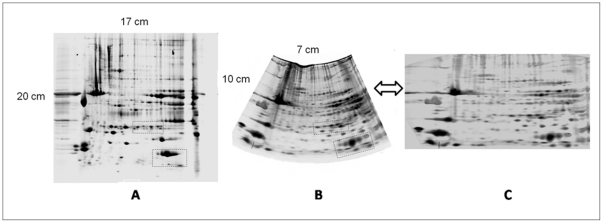
2-DE and 2-PE gel electrophoresis. Representative gel images obtained with 2-DE (panel A) and 2-PE (panel B). Panel C shows the radial map after the Delta2D assisted conversion of coordinates from polar to Cartesian. The lengths of the axes are reported. The highlighted areas of the gels are those reported in Figure 2 and 3 as enlarged sections. Sample load: 1.2 mg of total protein in A vs. 120 µg in B.

We named gels obtained by 2-DE “Cartesian set” and gels obtained by 2-PE “radial set”. A representative image for each set is shown in **Figure 1**. Radial gels should be analysed with the Delta2D software package. This software was previously upgraded with a specific algorithm for converting images from polar to Cartesian coordinates [6], since the Cartesian format is the more user friendly type of visualization. As in all 4^th^ generation software workflow, in Delta2D, spots are first warped to each other; thereafter, spot detection is performed only on an artificial “fusion gel”, creating a spot mask that is then overlaid on each warped gel image in the data set. Thus, using Delta2D, spot detection is performed only on the fusion image and, by definition, spot matching is always 100%. Conversely, our aim was to compare the number of detected spots in each gel and the matching efficiency obtained both with 2-DE and 2-PE. Therefore, although we consider Delta2D the most suitable software for the analysis of radial maps, for needs related to the experimental design of this study, we have used the PDQuest (version 7.3) software. In this study, a total of 23 replica gels have been analyzed for each dataset.

After matching the spots, the following parameters were taken into account to estimate gel-to-gel reproducibility among gels from each set: 1) efficiency of matching, defined as the percentage of matched spot on the average of detected spots between two gels, that is indicative of qualitative differences among gels; 2) coefficient of variation (CV) of matched spot intensities, that reflects the quantitative differences; 3) mean and CV of the total density in gel images.

The number of protein spots detected on 2-DE gels increases as the gel size increases. In fact, the trend observed over the years is the use of larger and larger 2-D gels. However, it has been experimentally demonstrated that, even in the largest-size 2-D gels now commercially available, multiple proteins are often visualized in the map as a single spot [9]. A possible solution would be to use giant gels, with a much increased resolution and capacity [10]–[13], but this technology is seldom used for technical problems related to the gel preparation and handling. Furthermore the use of giant gels implies loading of large quantities of protein extracts, which are difficult to obtain from most biological samples. Contrary to this trend of size increasing, the possibility to modify the shape of the 2-DE map has never been taken into account. In fact, since its inception, the two electrophoretic step of 2-DE were combined in a linear and orthogonal manner. Only recently, with the introduction of P-Dimensional electrophoresis [6], this canon was changed for the first time, with the application of a radial electric field that allows the migration of proteins from the first to the second dimension obtaining a gel map with a circular crown shape.

The first advantage of this approach regards the experimental reproducibility. The overall variability of 2-DE maps depends on sample and gel preparation, electrophoretic conditions, gel staining procedures [14] and even on quality of the software package used for the analysis [15]. Since IEF in IPGs is a highly reproducible steady state electrophoretic technique, the experimental variability of spot positions along the molecular mass axis is the major problem. In fact, differences in spot position are higher in the y-axis than in the x-axis, regardless of the use of different instrumentation to perform 2-DE [16]. A commonly used strategy to reduce this source of error is to use large SDS gels (24 cm) and load simultaneously more than one strip (e.g. three 7 cm long IPG strips) per gel. 2-D maps obtained with this approach make the subsequent analysis faster and more reliable, due to easier spot matching [17].

2-PE further ameliorates this multi-strip on one gel strategy: the radial shape of the gel can accommodate simultaneously up to six, 7-cm long strips along the inner circumference. In this report, the 23 radial maps (technical replicates) were obtained by four circular crown gels while the 23 Cartesian maps corresponded to 23 single gels.

Correlation analyses revealed a higher reproducible protein pattern from data obtained by 2-PE compared to 2-DE with a similar (not statistically significant) coefficient of variation of spot intensities but with a higher matching efficiency (+20%).

Another parameter to estimate the reproducibility of 2-DE and 2-PE is the measurement of the total density in gel images, which included both the intensities of the spots and of the background. We found that the mean and the CV of this value were significantly lower in the radial maps (mean: 1216×10^5^, CV: 24) respect to the Cartesian ones (mean: 1609×10^5^, CV: 46). We can confirm this result by observing the maps, where an even distribution of the background in radial maps can be appreciated. We can ascribe this result to the identical conditions of staining/destaining of radial maps. In fact, owing to the possibility to load up to six strips on the same radial gel, not only the migrations of proteins, but also the grayscales of the background are identical among the maps of the same gel. This is an important result, since in a proteomic study the accuracy in quantification for differentially expressed proteins will increase in absence of uneven backgrounds.

**Figure 2:**
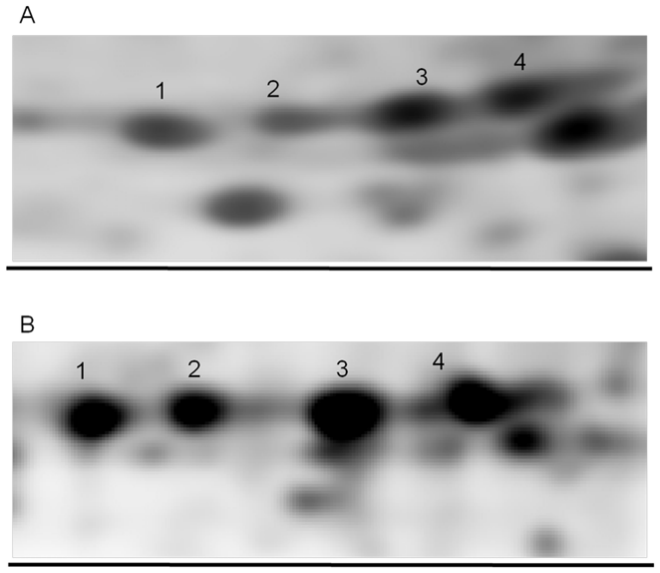
Enlarged sections of 2-DE and 2-PE gel images. Enlarged sections of gel images obtained with 2-DE (panel A) and 2-PE (panel B). The similar resolution of spots 1–4 in the two experimental sets can be visually appreciated. Protein spots 1–4 of 2-PE and 2-DE maps were identified by mass spectrometry analysis as different isoforms of triosephosphate isomerase (see Table S1 for MS protein identification parameters).

**Figure 3:**
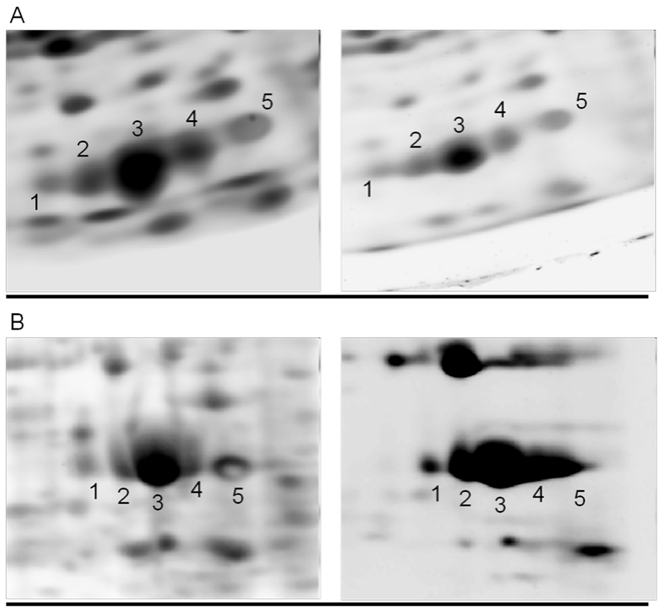
Enlarged sections of 2-DE and 2-PE gel images. Enlarged sections of gel images obtained with 2-DE (panel A) and 2-PE (panel B). The better resolution of spots 1–5 in the radial gel format can be visually appreciated. Protein spots 1–4 of 2-PE and 2-DE maps were identified by mass spectrometry analysis as different isoforms of myoglobin (see Table S1 for MS protein identification parameters).

Regarding the number of detected spots, results are much more complex to assess, since several variables come into play. The first variable regards the gel size. In this study, the Cartesian gels have an area that is approximately twice that of the radial gel. Radial and Cartesian maps were obtained using respectively 7 and 17 cm long strips. Despite the radial maps “started” with a disadvantage in terms of resolution in the first dimension, we observed that spots separation in the final map showed equivalent quality in the two sets, as can be appreciated in the example of **Figure 2** showing the separation of different isoforms of triosephosphate isomerase. Furthermore, in some instances, the migration of spots in the divergent radial field, despite of the reduced resolution related to the use of a shorter strip, was able to increase the resolution obtained in the Cartesian set, as can be observed in **Figure 3** for what concerning the myoglobin protein spots.

These cases were observed in the lower, fanning-out area of the map (see also **Figure 1**), where the effect of radial migration should theoretically lead to an accentuated spot resolution, directly proportional to the migration distance.

The second variable regards the protein loading. To make a fair comparison, we decided to load a higher amount of protein in the larger gel set, i.e. the Cartesian one. In fact, an advantage of using large gels, as well as the possibility to increase resolution, lies in the opportunity of increasing the loading of proteins thus bringing many more proteins above the detection limit and consequently increasing the number of detected spots. On the other hand, overloading would result in the risk of obscuring some zones, leading poor resolution in these zones. In this study, the absence of an overloading can be visually appreciated by looking at the **Figure 1**. Thanks to the increased loading of total protein (∼10×) in the Cartesian set, we observed the appearance of some spots (+10%), that were completely absent in the radial set.

**Figure 4:**
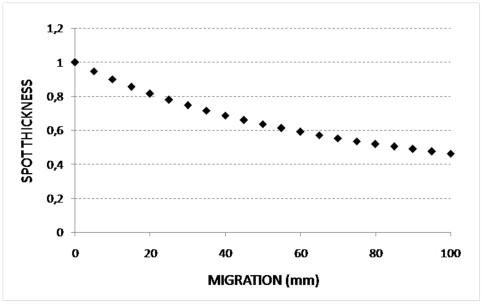
Spot thickness respect to the 2-PE migration. Plot of the thickness of a spot respect to the migration length in a radial electric field, showing that the spot thickness decreases with the progress of the migration.

The third variable concerns the presence of a gradient of porosity on the 2^nd^ dimension gel, which, for technical reasons, can be done only in Cartesian gels. Porosity gradients are useful because they give the highest possible resolution along the y-axis of the 2-DE map. However, in the radial maps we didn't observe any problem of resolution along the y-axis, such us vertical streaking made of stacked spots, despite the shorter run length (10 cm in the radial set *vs* 15 cm in the Cartesian set) and the lacking of the porosity gradient. We believe that this result could be due to an interesting side effect of the radial electric field geometry. In fact, in association with the distancing of spots during the electrophoretic migration, we observed also a flattening of the spots. Starting from these experimental observations, we made a theoretical model to further validate these findings. We approximate the spot shapes using circular sectors. Starting data are: 1) initial radii r_1,i_ and r_2,i_; 2) initial widths w_1,i_ and w_2,i_; 3) initial thicknesses t_1,i_ and t_2,i_. First of all, we can compute the angles *a_k_* by using the formula: “Equation 1”.
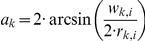
The spot areas, which are invariable during the run, can be found using the following formula “Equation 2”.
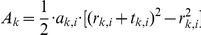
The width and the thickness of a spot can be modeled with respect to the time *s* using the following formula: “Equation 3”
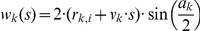
“Equation 4”
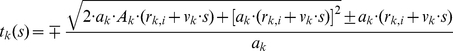
“Equation 4” was found by fixing the area of the spot in “Equation 2” and then solving it in t_k,i_. As far as “Equation 4” is concerned, we keep the sign combination that gives the positive result (since a real thickness cannot be negative). In this study, the radial gel had a inner radius of 7,5 cm and a outer radius of 17,5 cm. Starting from these dimensions, we calculated the spot thickness respect to the migration and found that the spot thickness decreases with the progress of the migration (**Figure 4**). We are currently carrying out further investigations to develop a more detailed mathematical model capable for representing the theoretical behavior of spot during polar electrophoresis, to find the ideal lengths of the radial gel radii and consequently to optimize the entire process. Thus, the observed slight increase in the number of spots in the Cartesian set depends only on the higher protein loading and not from differences in resolution. However, this increment was lower than what we would have expected on the basis of our experience with 2-DE. In fact, we have recently observed that, with a same protein load, a large gel (35×18) allows to visualize nearly twice as many spots in respect to a gel of smaller dimension (24×18) [18]. This discrepancy could be related to the gel shape, which in this study balanced the difference in gel size.

In conclusion, the gain in resolution obtained just by using a radial second-dimension gel can increase the resolution achieved during the IEF step. As a consequence, the comparative analysis reveals that, despite the smaller area, the absence of a gradient of porosity in the second dimension and the reduced loading, the radial set show a performance similar to the Cartesian one, but with the advantage of an associated increased reproducibility. Hence, for all the above-mentioned reasons, we retain that 2-PE appears an attractive innovation to further improve the performance of traditional two-dimensional gels.

## Materials and Methods

### Samples and Protein extraction

This methodological work has been based on samples analyzed for an ongoing study concerning the post-mortem proteome changes in bovine muscle. In particular the protein samples were obtained from *Longissimus dorsi* (LD) muscle of 8 different Charolais bovines.

Approximately 200 mg (wet weight) of each muscle biopsy was homogenized in 1500 µl of solubilising/lysing solution [7 M urea (Sigma, Sigma-Aldrich Corporation, St. Louis, MO, USA), 2 M thiourea (Sigma), 3% CHAPS (Sigma), 20 mM Tris (Sigma), and 1× protease inhibitor cocktail tablet (Complete, Mini; Roche, Basel, Switzerland)] with an ultraturrax (Polytron PT, Kinematica AG, Luzern Switzerland) at 12000 rpm for 5×30 s. The homogenate was precipitated twice with 4 volumes of acetone and resuspended in 600 µl of solubilising/lysing solution. Then 1% pH 3–10 Ampholyte was added and samples were centrifuged for 40 min at 14,000× g at 4°C to remove the complexed nucleic acids. Protein extracts were incubated with 5 mM tributyl phosphine and 10 mM acrylamide for 90 minutes at room temperature to reduce protein disulphide bonds and alkylate the cysteine thiolic groups. The reaction was blocked by the addition of 10 mM DTT (Sigma) and the samples were collected and stored at −80°C. Protein concentrations were measured with a commercial kit at 750 nm (DC Protein Assay, Bio-Rad) using BSA as standard.

### 2-DE Gel Electrophoresis

Protein fractionation by 2-DE was performed as previously described [7]. Briefly, 400 µl of each sample (containing 1.2 mg of total protein) was separated in 17 cm long pH 3–10 NL IPG strips, and the total product time×voltage applied was 75000 Vh for each strip. The second dimensional separation was done using 8–18%T gradient SDS-PAGE, applying 40 mA for each gel for 3 min, then 2 mA/gel for 1 h, and 20 mA/gel until the tracking dye, Bromophenol-Blue, reached the anodic end of the gels.

### 2-PE Gel Electrophoresis

150 µl of each sample (containing 120 µg of total protein) was separated by 7 cm long pH 3–10 NL IPG strip, and the total product time×voltage applied was 25000 Vh for each strip. The IPG strips were then transferred onto a 12%T circular crown gel and the second separation was performed in a radial electrophoretic chamber (Elettrofor SAS, Borsea, Rovigo, Italy). Runs were carried out at 50 V until the bromophenol blue reached the bottom of the gel.

### Gel Visualization, Image Acquisition, and Analysis

After 2-DE and 2-PE, proteins were detected by Sypro Ruby. Gels were incubated in a fixing solution containing 40% ethanol and 10% acetic acid for 30 minutes followed by overnight staining in a ready-to-use Sypro Ruby solution. Destaining was performed in 10% methanol and 7% acetic acid for 1 h, followed by a rinse of at least 3 hrs in pure water.

The image analysis of all the gel replicas was performed by PDQuest version 7.3. Each gel was analysed for spot detection, background subtraction and protein spot OD intensity quantification. The gel image showing the higher number of spots and the best protein pattern was chosen as a reference template, and spots in a standard gel were then matched across all gels. For both 2-DE and 2-PE analysis, the spots were matched to all replicated gel, and the variance coefficient in each dataset determined.

### Mass spectrometry and protein identification

Protein identification was performed after in-gel trypsin digestion as previously described [8]. Briefly, peptides from 5 µl of each sample were then separated by reversed phase nano-HPLC-Chip technology (Agilent Technologies, Palo Alto, CA, USA) online-coupled with a 3D ion trap mass spectrometer (model Esquire 6000, Bruker Daltonics, Bremen, Germany). Database searches were conducted using the MS/MS ion search of Mascot against all entries of the non-redundant NCBI database with the following parameters: up to one missed cleavage; variable modifications: propionamide (Cys) and oxidation (Met); peptide and fragment tolerances: ±0.9 Da and ±0.9 Da, respectively. For positive identification, the score of the result of [−10×Log(P)] had to be over the significance threshold level (p<0.01) and at least 2 different peptides (p<0.05) had to be assigned.

## Supporting Information

Table S1 **The identified proteins corresponding to spots indicated in** **Figure 2** **and** **3** **, together with the identification parameters (NCBInr accession number, number of identified peptides, Mascot score, theoretical Mr and pI, and the sequence coverage).**
(DOC)
