# Supplementary material for: Polar Electrophoresis: Shape of Two-Dimensional Maps Is as Important as Size
Source: PLoS One. 2012 Jan 23;7(1):e30911. doi: 10.1371/journal.pone.0030911 (PMC3264644; doi:10.1371/journal.pone.0030911)
Supplement: Table S1 — The identified proteins corresponding to spots indicated in Figure 2 and 3 , together with the identification parameters (NCBInr accession number, number of identified peptides, Mascot score, theoretical Mr and pI, and the sequence coverage). (DOC) [file pone.0030911.s001.doc]

**Figure 2:**

| **Protein name** | **Spot no.** | **NCBI**  **acc. #** | **No. of peptides identified** | **Mascot score** | **Mr. (Da) theor.** | **pI theor.** | **Sequence Coverage (%)** |
| --- | --- | --- | --- | --- | --- | --- | --- |
| **2-PE :** |  |  |  |  |  |  |  |
| triosephosphate isomerase | 1 | [gi|61888856](http://www.matrixscience.com/cgi/protein_view.pl?file=../data/20110705/FttriaTnR.dat&hit=gi|61888856&db_idx=1&px=1&ave_thresh=48&_ignoreionsscorebelow=0&report=0&_sigthreshold=0.05&_msresflags=1025&_msresflags2=2&percolate=-1&percolate_rt=0) | 5 | 237 | 26673 | 6.45 | 18 |
| triosephosphate isomerase | 2 | [gi|61888856](http://www.matrixscience.com/cgi/protein_view.pl?file=../data/20110705/FttriaTnR.dat&hit=gi|61888856&db_idx=1&px=1&ave_thresh=48&_ignoreionsscorebelow=0&report=0&_sigthreshold=0.05&_msresflags=1025&_msresflags2=2&percolate=-1&percolate_rt=0) | 10 | 224 | 26673 | 6.45 | 18 |
| triosephosphate isomerase | 3 | [gi|61888856](http://www.matrixscience.com/cgi/protein_view.pl?file=../data/20110705/FttriaTnR.dat&hit=gi|61888856&db_idx=1&px=1&ave_thresh=48&_ignoreionsscorebelow=0&report=0&_sigthreshold=0.05&_msresflags=1025&_msresflags2=2&percolate=-1&percolate_rt=0) | 10 | 339 | 26673 | 6.45 | 16 |
| triosephosphate isomerase | 4 | [gi|61888856](http://www.matrixscience.com/cgi/protein_view.pl?file=../data/20110705/FttriaTnR.dat&hit=gi|61888856&db_idx=1&px=1&ave_thresh=48&_ignoreionsscorebelow=0&report=0&_sigthreshold=0.05&_msresflags=1025&_msresflags2=2&percolate=-1&percolate_rt=0) | 10 | 251 | 26673 | 6.45 | 17 |
| **2-DE :** |  |  |  |  |  |  |  |
| triosephosphate isomerase | 1 | [gi|61888856](../../../../E:%5CMASCOT%20spectra%20%28.mgf%29%5CIDENTIFICAZIONI%20RIUSCITE%5CMichele%20Menini%5CCARTESIANI%5C4204%20master_results.pl#Hit1) | 14 | 391 | 26673 | 6.45 | 23 |
| triosephosphate isomerase | 2 | [gi|61888856](http://www.matrixscience.com/cgi/protein_view.pl?file=../data/20110705/FttriaTah.dat&hit=gi|61888856&db_idx=1&px=1&ave_thresh=47&_ignoreionsscorebelow=0&report=0&_sigthreshold=0.05&_msresflags=1025&_msresflags2=2&percolate=-1&percolate_rt=0) | 10 | 288 | 26673 | 6.45 | 19 |
| triosephosphate isomerase | 3 | [gi|61888856](http://www.matrixscience.com/cgi/protein_view.pl?file=../data/20110705/FttriaTmh.dat&hit=gi|61888856&db_idx=1&px=1&ave_thresh=47&_ignoreionsscorebelow=0&report=0&_sigthreshold=0.05&_msresflags=1025&_msresflags2=2&percolate=-1&percolate_rt=0) | 15 | 396 | 26673 | 6.45 | 19 |
| triosephosphate isomerase | 4 | [gi|61888856](http://www.matrixscience.com/cgi/protein_view.pl?file=../data/20110705/FttriaTmh.dat&hit=gi|61888856&db_idx=1&px=1&ave_thresh=47&_ignoreionsscorebelow=0&report=0&_sigthreshold=0.05&_msresflags=1025&_msresflags2=2&percolate=-1&percolate_rt=0) | 10 | 258 | 26673 | 6.45 | 16 |

**Figure 3:**

| **Protein name** | **Spot no.** | **NCBI**  **acc. #** | **No. of peptides identified** | **Mascot score** | **Mr. (Da) theor.** | **pI theor.** | **Sequence Coverage (%)** |
| --- | --- | --- | --- | --- | --- | --- | --- |
| **2-PE :** |  |  |  |  |  |  |  |
| myoglobin | 1 | [gi|27806939](../../../../E:%5CMASCOT%20spectra%20%28.mgf%29%5CIDENTIFICAZIONI%20RIUSCITE%5CMichele%20Menini%5CCARTESIANI%5C6112%20master_results.pl#Hit1) | 4 | 112 | 17067 | 6.90 | 18 |
| myoglobin | 2 | [gi|230253](http://www.matrixscience.com/cgi/protein_view.pl?file=../data/20110706/Fttribeam.dat&hit=gi|230253&db_idx=1&px=1&ave_thresh=48&_ignoreionsscorebelow=0&report=0&_sigthreshold=0.05&_msresflags=1025&_msresflags2=2&percolate=-1&percolate_rt=0) | 5 | 125 | 16943 | 6.83 | 20 |
| myoglobin | 3 | [gi|230253](http://www.matrixscience.com/cgi/protein_view.pl?file=../data/20110706/Fttribete.dat&hit=gi|230253&db_idx=1&px=1&ave_thresh=47&_ignoreionsscorebelow=0&report=0&_sigthreshold=0.05&_msresflags=1025&_msresflags2=2&percolate=-1&percolate_rt=0) | 6 | 154 | 16943 | 6.83 | 20 |
| myoglobin | 4 | [gi|27806939](http://www.matrixscience.com/cgi/protein_view.pl?file=../data/20110706/FttribTSm.dat&hit=gi|27806939&db_idx=1&px=1&ave_thresh=47&_ignoreionsscorebelow=0&report=0&_sigthreshold=0.05&_msresflags=1025&_msresflags2=2&percolate=-1&percolate_rt=0) | 5 | 120 | 17067 | 6.90 | 20 |
| myoglobin | 5 | [gi|118595824](http://www.matrixscience.com/cgi/protein_view.pl?file=../data/20110610/FtteSesEh.dat&hit=gi|118595824&db_idx=1&px=1&ave_thresh=47&_ignoreionsscorebelow=0&report=0&_sigthreshold=0.05&_msresflags=1025&_msresflags2=2&percolate=-1&percolate_rt=0) | 4 | 69 | 17369 | 8.98 | 11 |
| **2-DE :** |  |  |  |  |  |  |  |
| myoglobin | 1 | [gi|27806939](../../../../E:%5CMASCOT%20spectra%20%28.mgf%29%5CIDENTIFICAZIONI%20RIUSCITE%5CMichele%20Menini%5CCARTESIANI%5C6112%20master_results.pl#Hit1) | 3 | 59 | 17067 | 6.90 | 26 |
| myoglobin | 2 | [gi|230253](http://www.matrixscience.com/cgi/protein_view.pl?file=../data/20110705/FttriaTEm.dat&hit=gi|230253&db_idx=1&px=1&ave_thresh=48&_ignoreionsscorebelow=0&report=0&_sigthreshold=0.05&_msresflags=1025&_msresflags2=2&percolate=-1&percolate_rt=0) | 6 | 153 | 16943 | 6.83 | 20 |
| myoglobin | 3 | [gi|27806939](http://www.matrixscience.com/cgi/protein_view.pl?file=../data/20110705/FttriaTwE.dat&hit=gi|27806939&db_idx=1&px=1&ave_thresh=47&_ignoreionsscorebelow=0&report=0&_sigthreshold=0.05&_msresflags=1025&_msresflags2=2&percolate=-1&percolate_rt=0) | 17 | 336 | 17067 | 6.90 | 32 |
| myoglobin | 4 | [gi|27806939](../../../../E:%5CMASCOT%20spectra%20%28.mgf%29%5CIDENTIFICAZIONI%20RIUSCITE%5CMichele%20Menini%5CCARTESIANI%5C7114%20master_results.pl#Hit1) | 9 | 151 | 17067 | 6.90 | 20 |
| myoglobin | 5 | [gi|27806939](../../../../E:%5CMASCOT%20spectra%20%28.mgf%29%5CIDENTIFICAZIONI%20RIUSCITE%5CMichele%20Menini%5CCARTESIANI%5C7114%20master_results.pl#Hit1) | 4 | 116 | 17067 | 6.90 | 20 |
